# Supplementary material for: Mapping the glycosyltransferase fold landscape using interpretable deep learning
Source: Nat Commun. 2021 Sep 27;12:5656. doi: 10.1038/s41467-021-25975-9 (PMC8476585; doi:10.1038/s41467-021-25975-9)
Supplement: Supplementary file 1 — Supplementary Information [file 41467_2021_25975_MOESM1_ESM.pdf]

Supplementary Information

Supplementary Figures

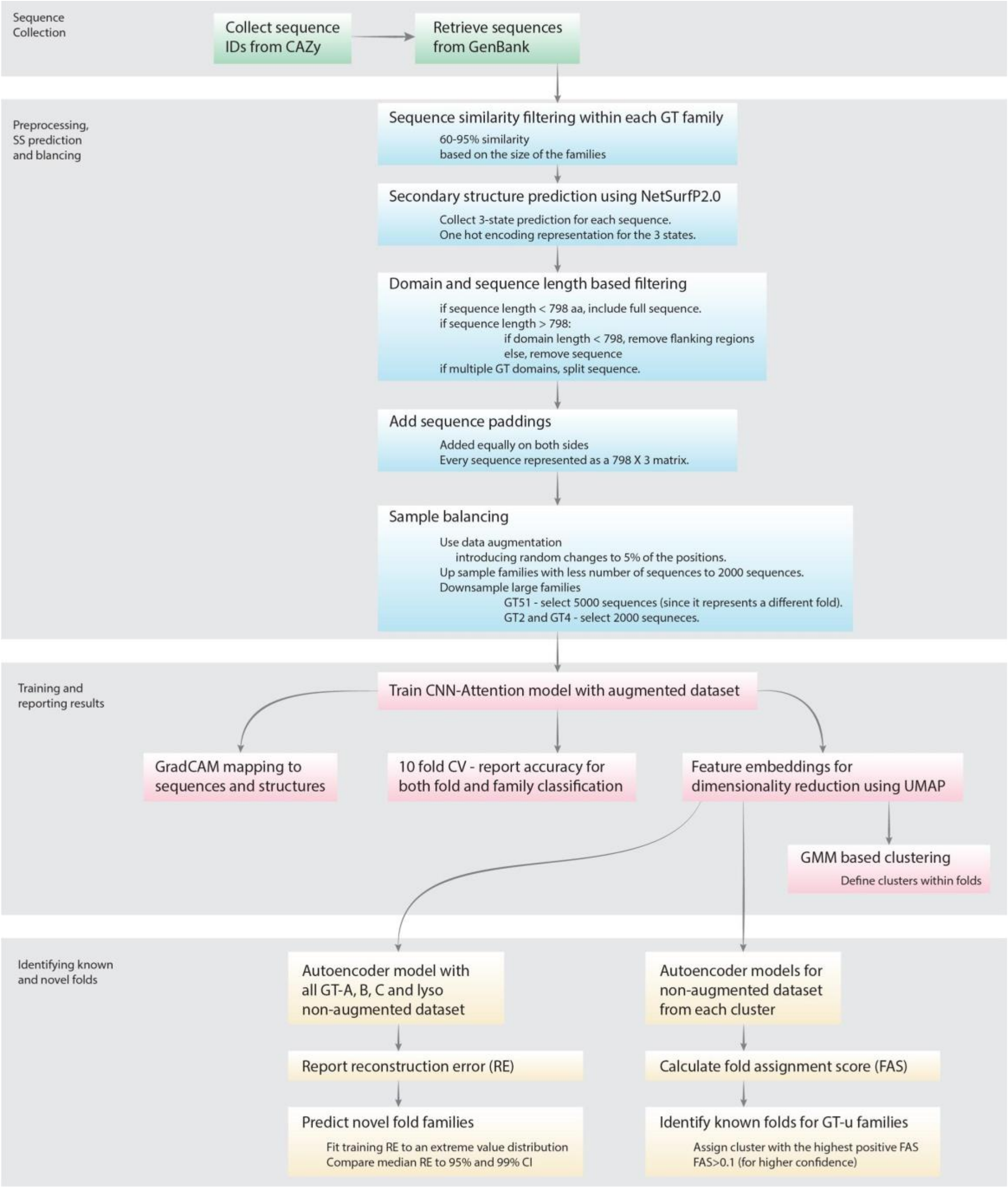

Supplementary Figure 1: Flowchart showing the preprocessing, training and interpretation steps of the CNN-Attention and autoencoder model.

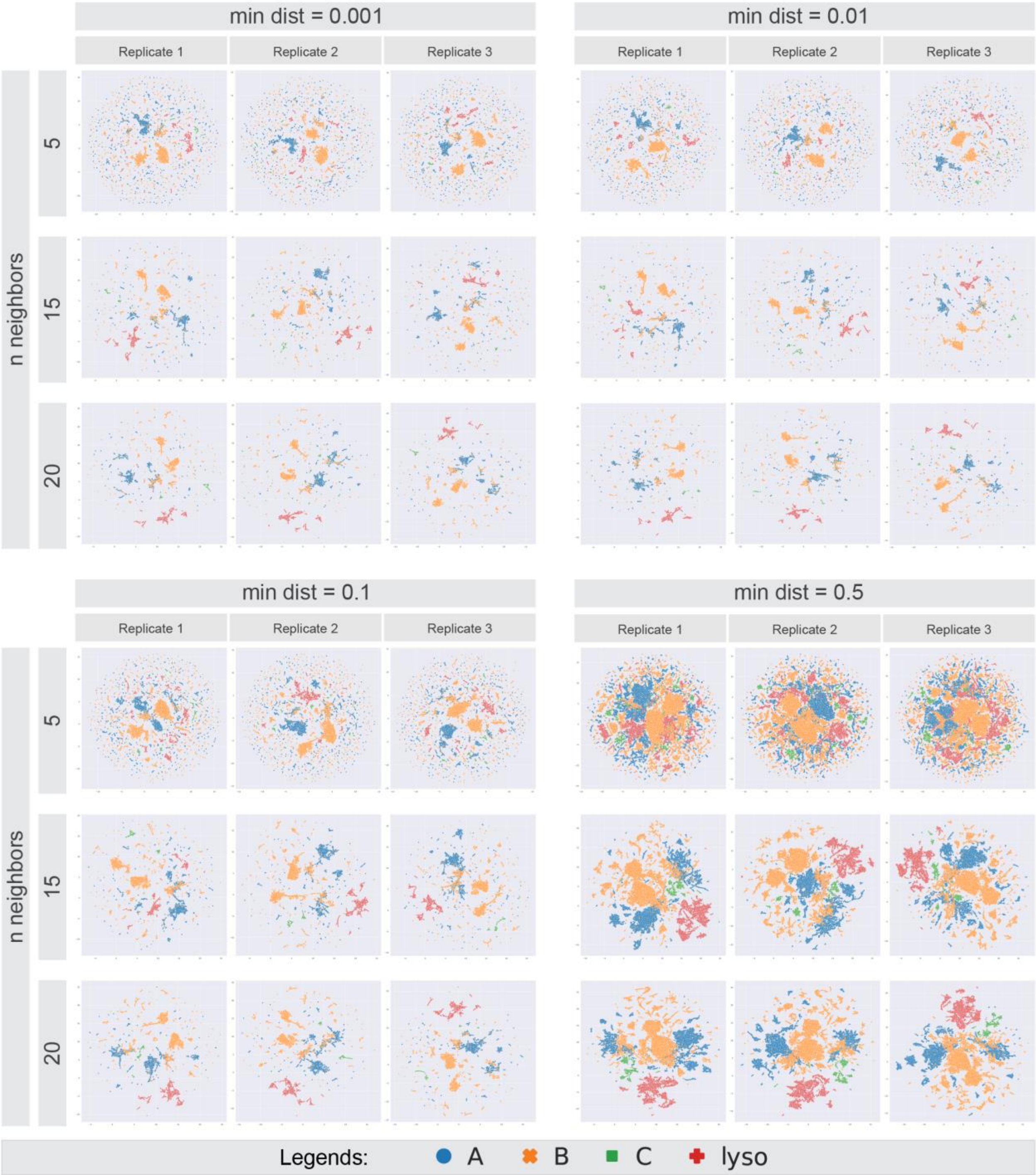

Supplementary Figure 2: Scan of UMAP Projections across different parameters using the transformer based Esm-1b embeddings. The projections are generated from the embeddings of all trained GT sequences obtained using the transformer based esm-1b method. Following the esm-1b instructions, the mean values are taken across all positions to generate a vector of 1280 dimensions. UMAP was applied to this high dimensional data to generate these two dimensional projections for visualization of GT fold clusters. 3 replicate projections of each combination of parameters are obtained to ensure reproducibility.

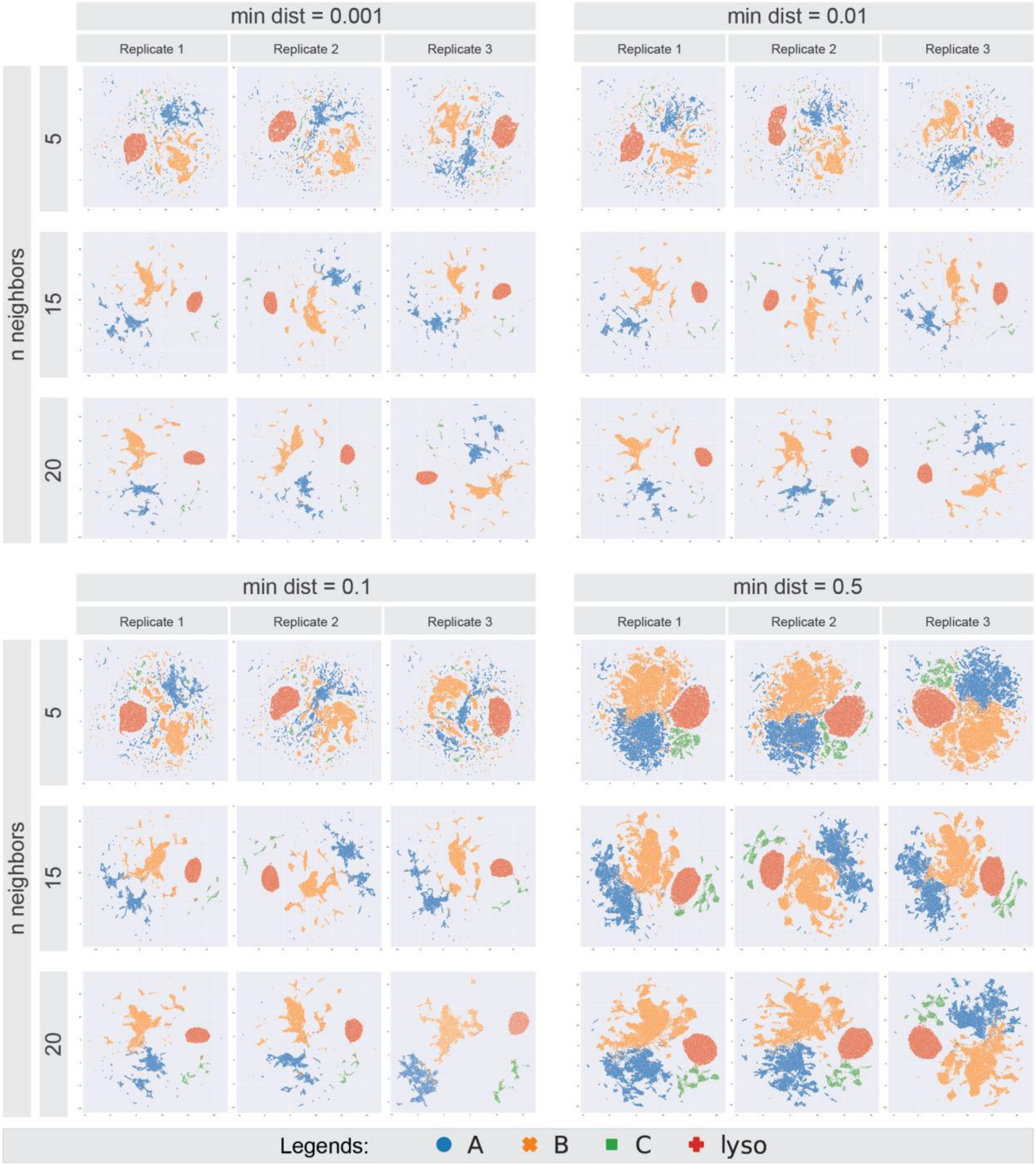

Supplementary Figure 3: Scan of UMAP Projections across different parameters using CNN-attention embeddings. The projections are generated from the embeddings of all trained GT sequences obtained using the GT-CNN method. 3 replicate projections of each combination of parameters are obtained to ensure reproducibility. The UMAP projection using the parameters (n_neighbors =20, min_dist =0.1) was selected as the final projection.

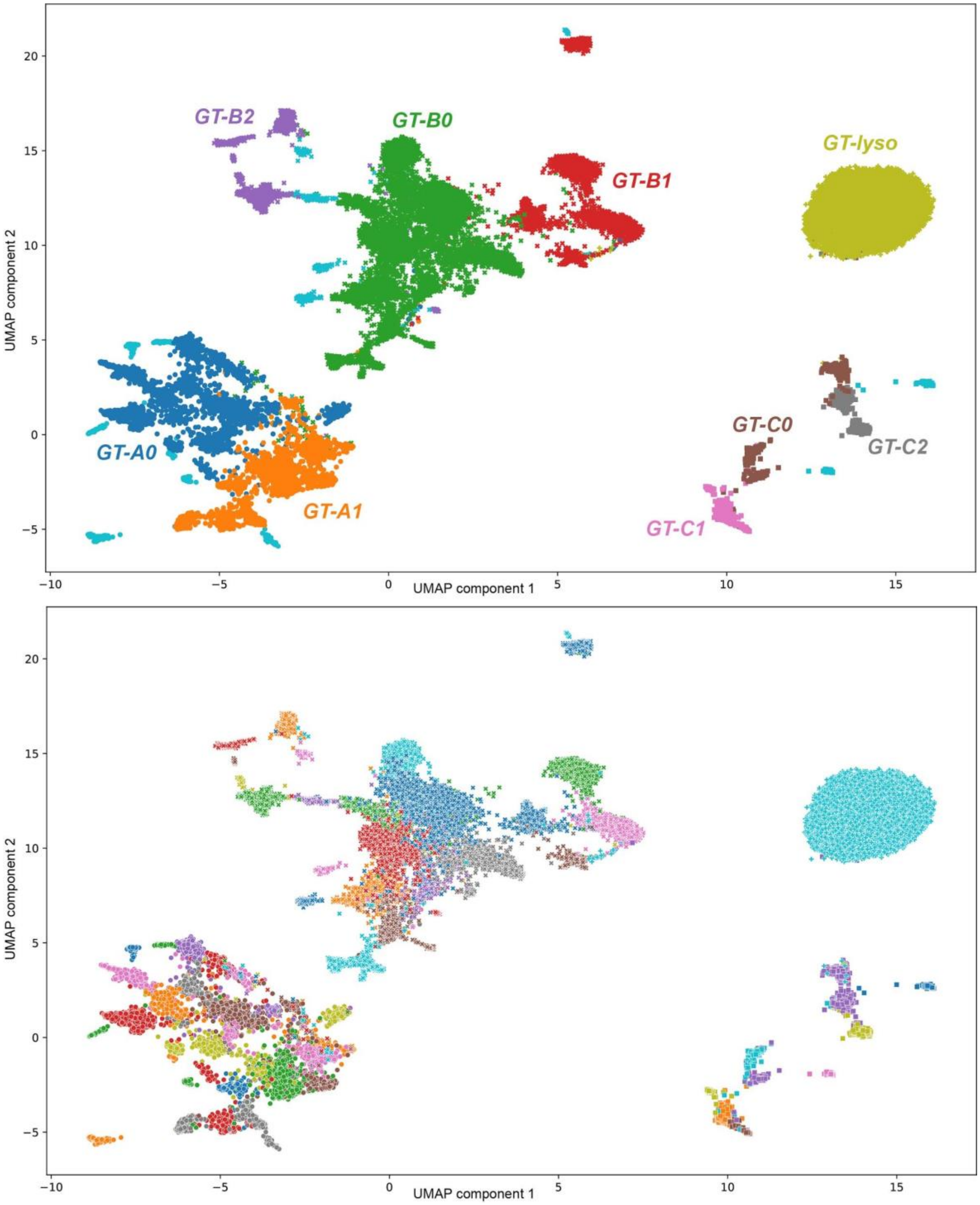

Supplementary Figure 4: UMAP projections for the feature vectors of the major GT fold types. Sequences are colored based on their cluster assignments (Top) and based on CAZy families (Bottom). Both plots show sequences from the same cluster and family grouping together, respectively.

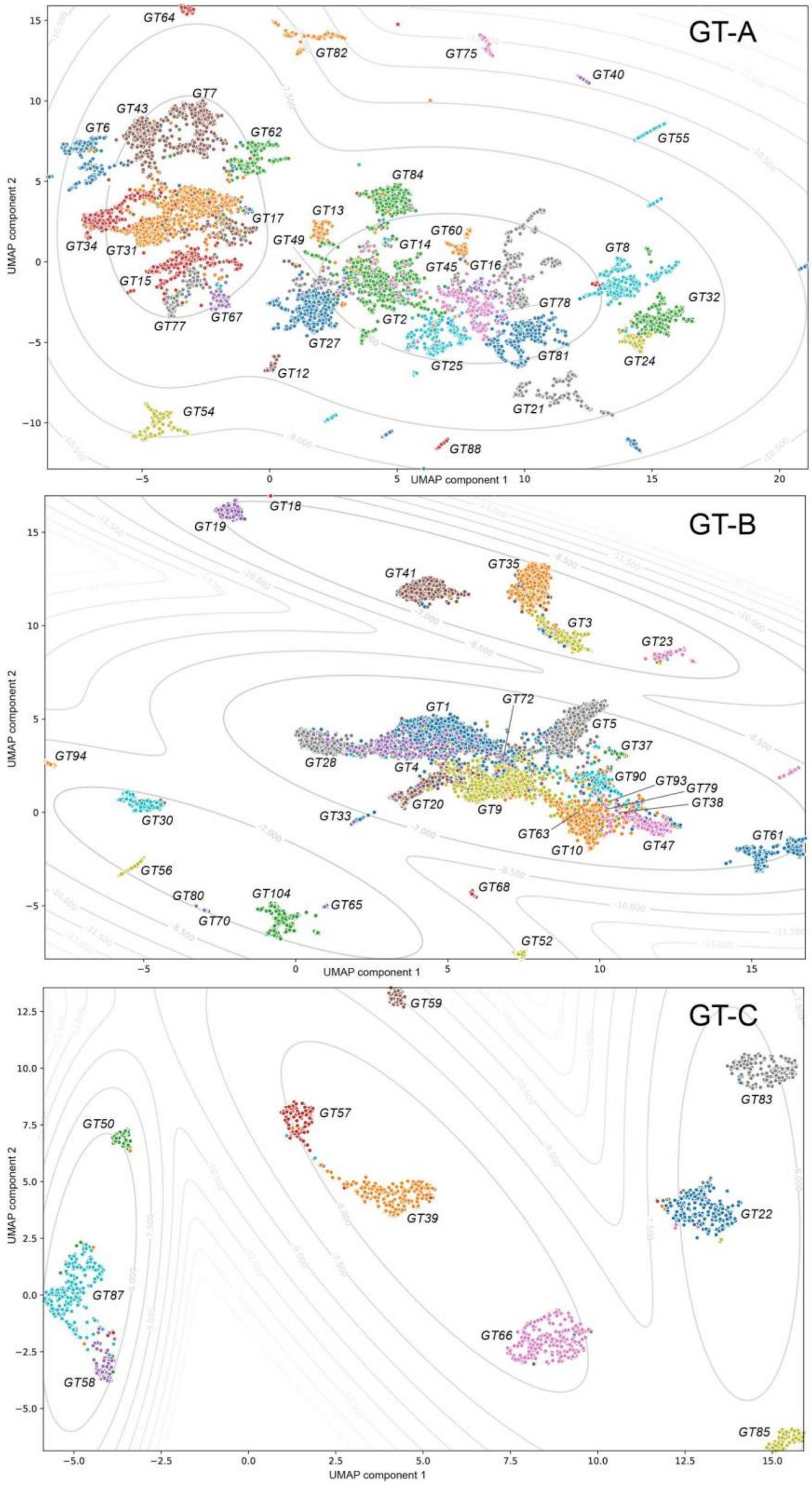

Supplementary Figure 5: UMAP projections for the feature vectors from separate fold types. Sequences are colored based on their family and labelled.

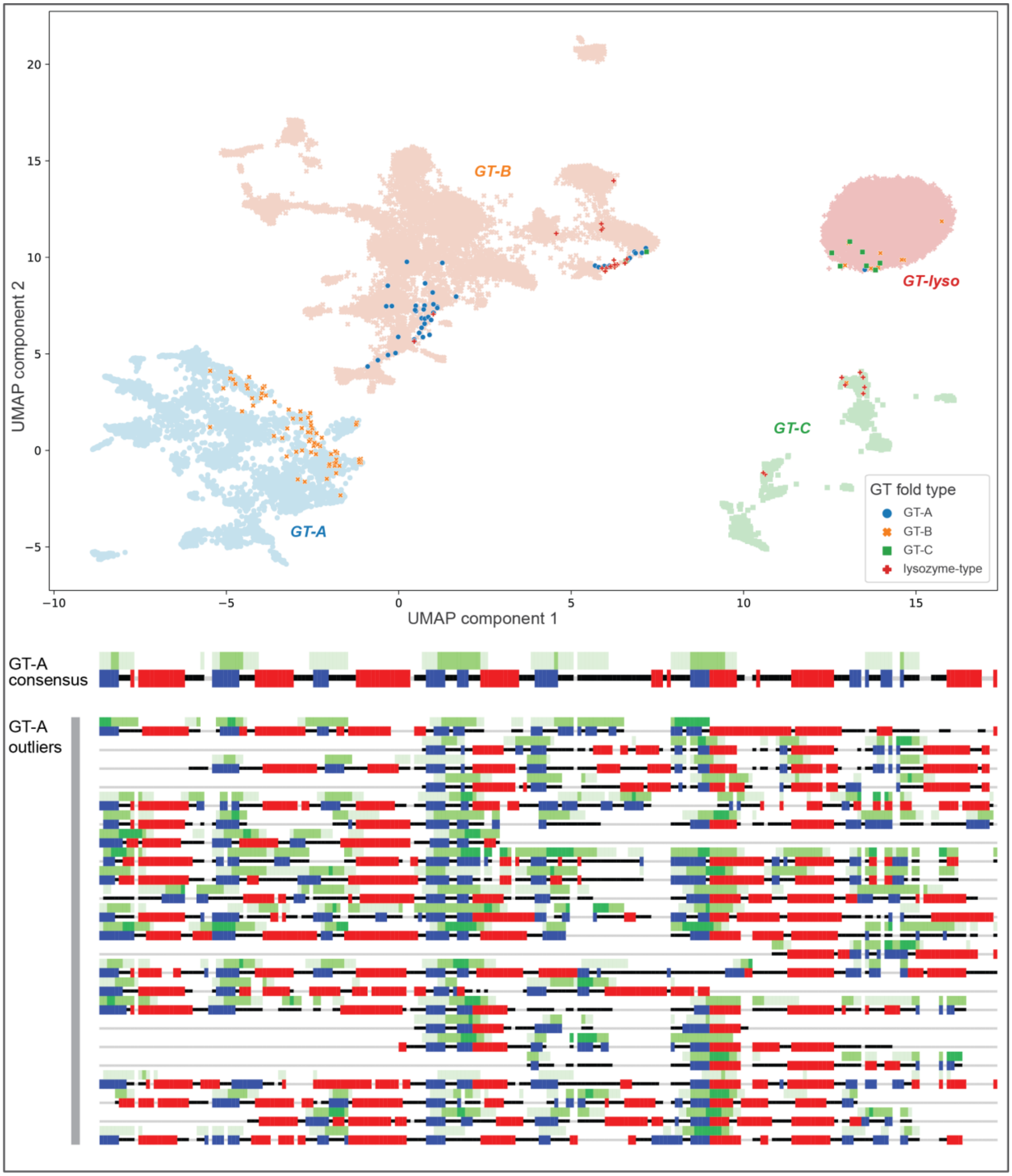

Supplementary Figure 6: Analysis of the outlier sequences. Top: Outlier sequences are highlighted in the UMAP projections. Most of these sequences include fragmentary sequences. Bottom: Layer 2 CAM maps for the aligned GT-A domain of the outlier sequences compared to the GT-A consensus. Other folds are not shown since there is no fold level consensus alignment for comparison.

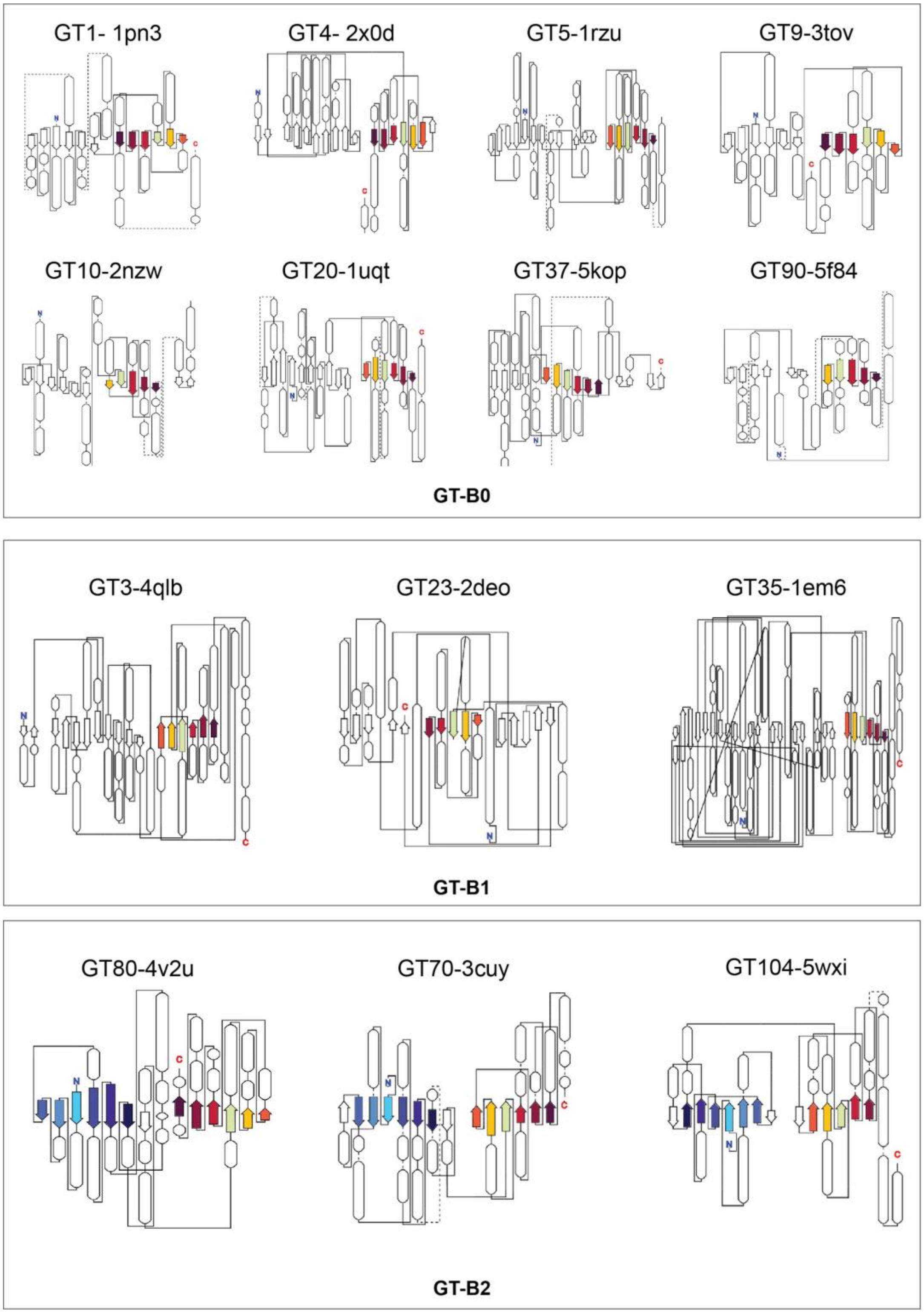

Supplementary Figure 7: Topologies for multiple representative structures for GT-B families from different clusters show the core conserved features identified by the CNN-Attention module.

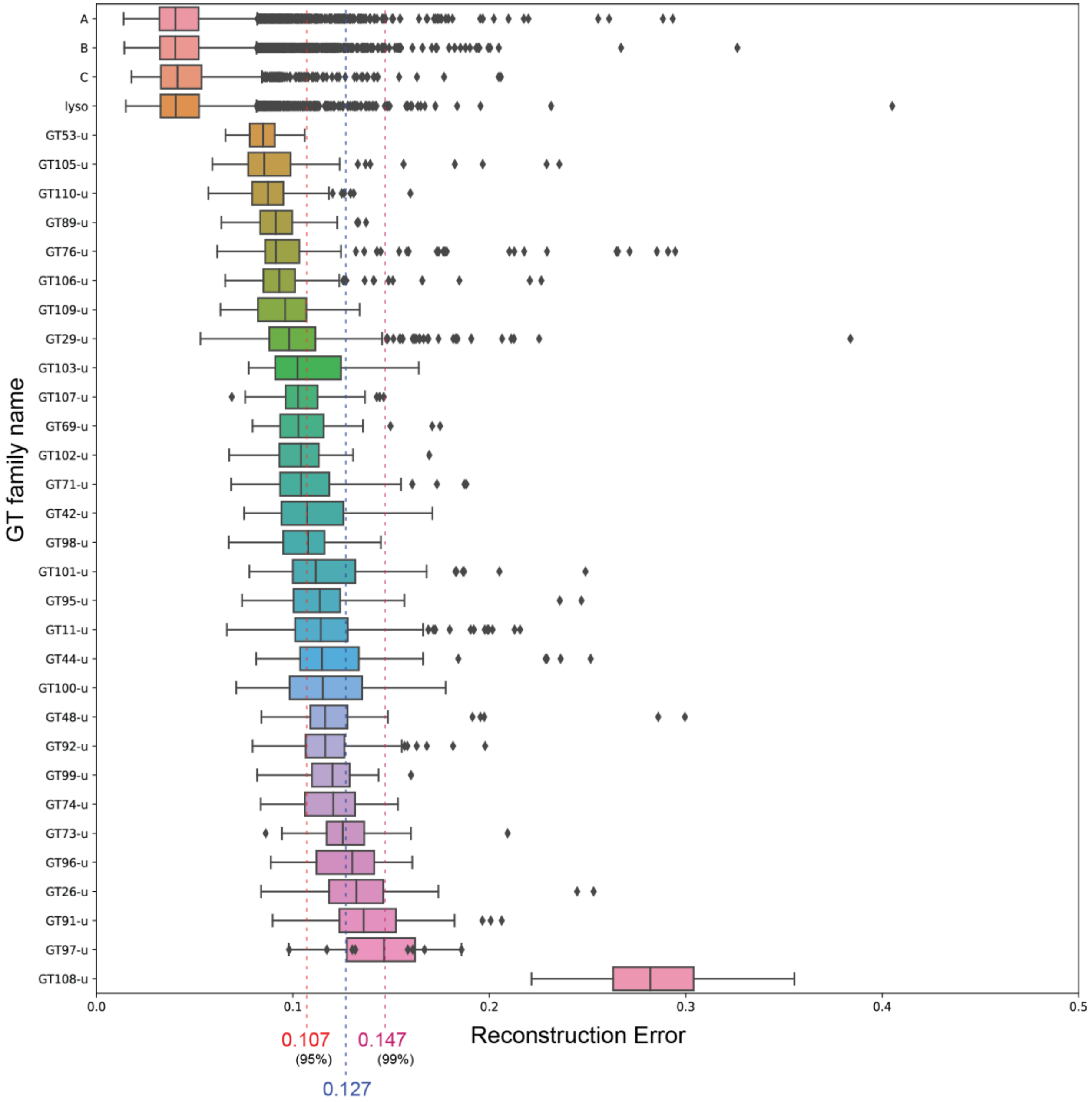

Supplementary Figure 8: Boxplot showing the RE for each of the 4 known GT folds and all the GT-u families. The ends of boxes show the 1st and the 3rd quartiles and the line within the box indicates the median value while the whiskers mark the 1.5 times the interquartile range excluding the outliers shown as individual diamonds. The red line at 0.104 indicates the 95% CI upper bound based on the extreme value distribution of the training (known GT folds) sequences. Magenta line at 0.147 indicates the 99% CI upper bound. These CI were derived by fitting an extreme value distribution to the REs of GT-A, -B, -C and -lyso sequences of n=24892 sequences. Blue line at 0.127 indicates the mid-point. GT-u families with median RE above this value are predicted to have novel GT folds with increasing confidence. Source data are provided as a Source Data file.

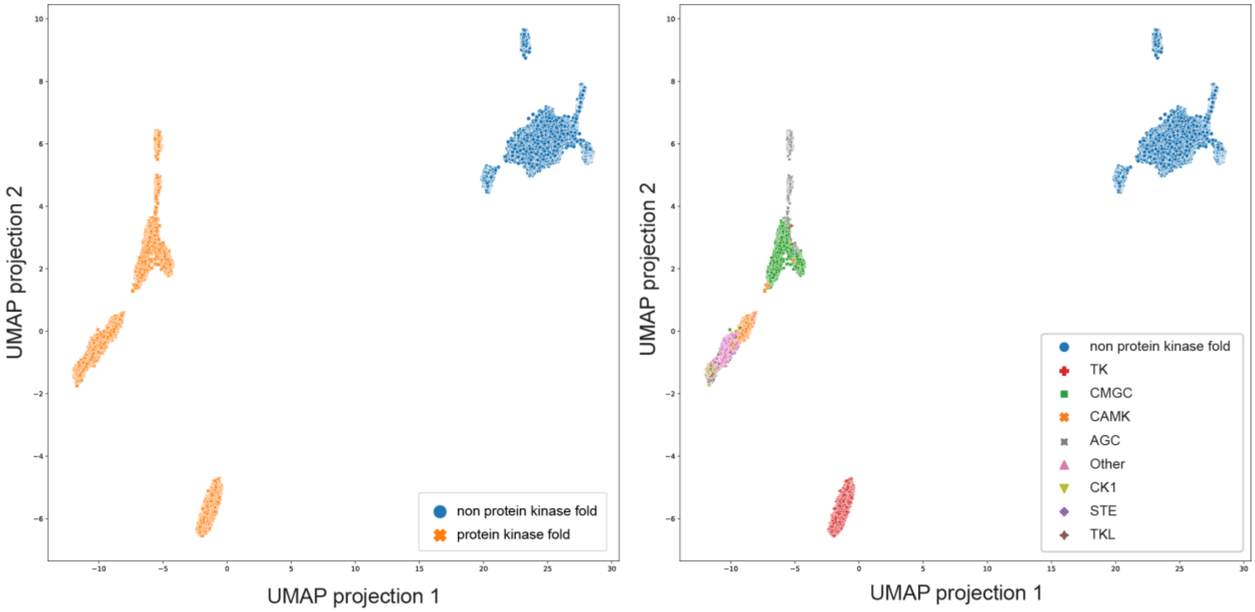

Supplementary Figure 9: UMAP projections obtained from the CNN-attention model trained on 11253 kinase sequences. The 2D projections show a clear separation of protein kinase fold sequences from non-protein kinase fold sequences (left). Separation also places kinases from the same group together (right). The accuracy for protein kinase fold separation from non-protein kinase fold is 99%. The accuracy for the separation of kinase groups is 83%. Source data are provided as a Source Data file.

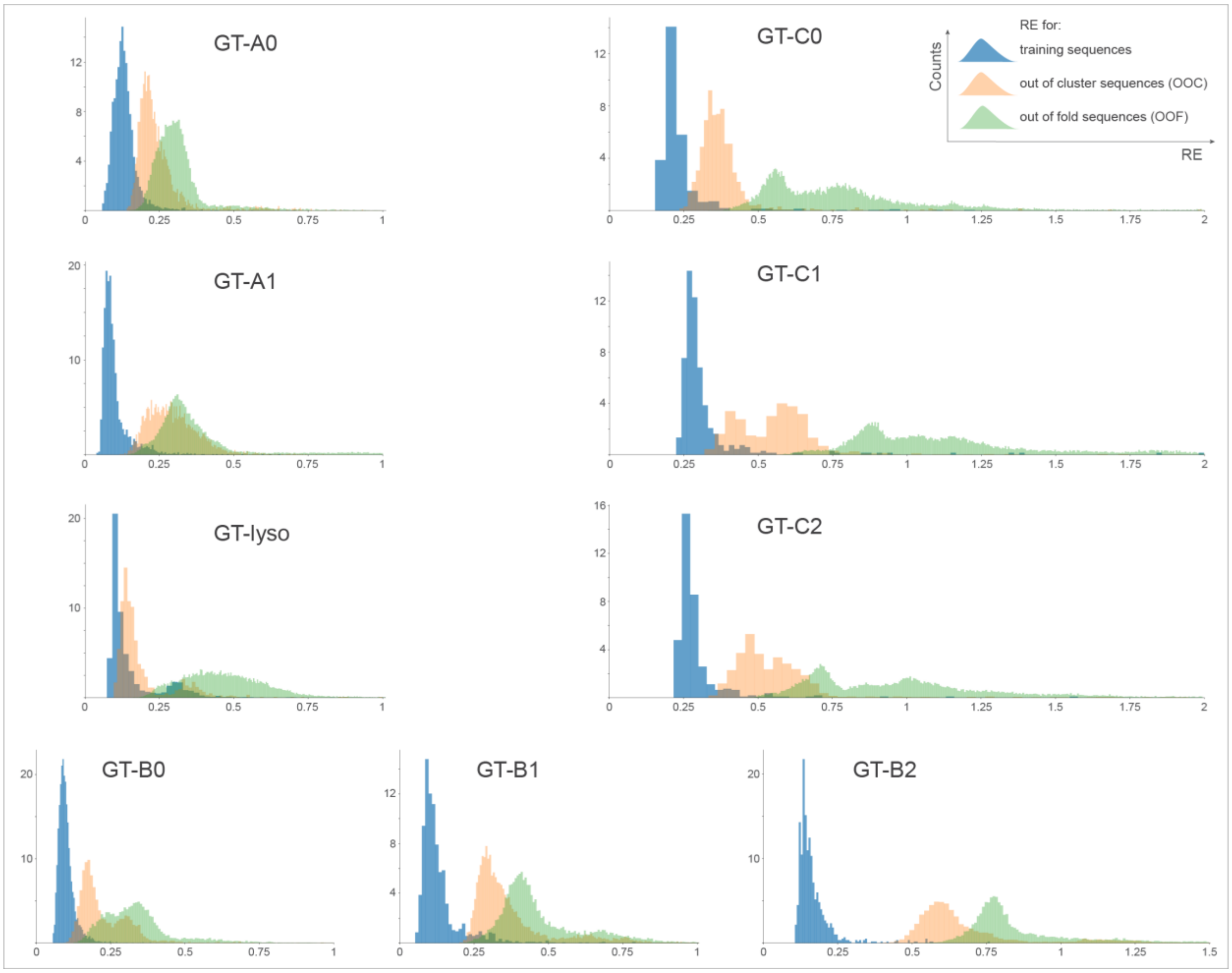

Supplementary Figure 10: RE distribution for the training, out of cluster and out of fold sequences for each of the 9 clusters generated using the cluster specific autoencoder models. Source data are provided as a Source Data file.

Supplementary Tables

Supplementary Table 1: A complete comparison of different modules in the CNN-attention model. The model was trained with the same hyperparameter settings: learning rate at 5e-5 and weight decay rate at 1e-5 using Adam optimizer. Datasets were separated as augmented (Aug) or non-augmented (Non-Aug) to make comparison of the effect of the data augmentation method. The effects of multitask learning and attention modules were also tested.

Model	Dataset	Target	Precision	Recall	Accuracy	F1-score
CNN+ Multitask	Non-Aug	Fold	0.85	0.87	0.87	0.86
		Family	0.23	0.29	0.29	0.23
CNN+ Multitask+ Attention	Non-Aug	Fold	0.90	0.90	0.90	0.89
		Family	0.29	0.35	0.35	0.29
CNN+ Multitask	Aug	Fold	0.92	0.92	0.92	0.92
		Family	0.59	0.57	0.57	0.55
CNN+ Attention	Aug	Fold	0.96	0.96	0.96	0.96
		Family	0.72	0.69	0.69	0.69
CNN+ Multitask+ Attention	Aug	Fold	0.96	0.96	0.96	0.96
		Family	0.78	0.77	0.77	0.77

Supplementary Table 2: Comparison of the prediction results from the GT CNN-attention model and the HHsearch results. The best 3 hits from the HHsearch results with an e-value lower than 1e-2 is considered for fold assignment using HHsearch. Whether HHsearch results match the CNN-attention network and/or the results from previous literature is also indicated.

GT-u fam	GT CNN-attention prediction	Literature support	HHsearch	Does HHsearch results match GT-CNN and literature support?	HHsearch results					
					1st best hit	E-val	2nd best hit	E-val	3rd best hit	E-val
GT100-u	variant	-	GT-B	No	GT52-B	6.21E-11	GT38-B	1.41E-08	GT80-B	1.00E-07
GT101-u	variant	variant	GT-A	No	GT2-A	7.06E-16	GT45-A	2.08E-13	GT8-A	8.14E-11
GT102-u	GT-B	-	none	No	GT5-B	2.52E-01	GT5-B	4.49E-01	GT63-B	5.22E-01
GT103-u	GT-B	-	Both A and B	No	GT25-A	9.45E-11	GT4-B	1.61E-06	GT4-B	5.93E-02
GT105-u	GT-C	GT-C	GT-B	No	GT41-B	3.12E-30	GT41-B	5.91E-25	GT41-B	1.40E-19
GT106-u	GT-B	-	GT-B	Yes	GT65-B	3.89E-06	GT68-B	1.26E-04	GT23-B	1.38E-04
GT107-B	GT-B	-	GT-B	Yes	GT19-B	3.57E-03	GT4-B	3.76E-02	GT41-B	1.11E-01
GT108-u	novel	novel	none	Yes	GT4-B	3.62E+00	GT56-B	4.44E+00	GT35-B	5.86E+00
GT109-u	GT-A	GT-A	GT-A	Yes	GT54-A	7.28E-21	GT25-A	3.09E-06	GT13-A	2.65E-03
GT110-u	GT-A	-	GT-B	No	GT47-B	1.19E-06	GT10-B	3.89E-03	GT5-B	1.70E-01
GT11-u	variant	-	GT-B	No	GT23-B	1.60E-10	GT68-B	6.31E-04	GT65-B	8.56E-04
GT26-u	novel	novel	none	Yes	GT5-B	1.03E-02	GT41-B	6.27E-01	GT30-B	1.14E+00
GT29-u	GT-A	GT-A variant	none	No	GT56-B	8.01E+00	GT52-B	9.20E+00	GT20-B	1.03E+01
GT42-u	variant	GT-A variant	none	Yes	GT70-B	1.71E+00	GT57-C	1.94E+00	GT52-B	2.42E+00
GT44-u	variant	-	GT-A	No	GT32-A	2.58E-11	GT88-A	3.55E-04	GT63-B	2.76E-01
GT48-u	variant	-	none	Yes	GT10-B	3.10E+00	GT27-A	8.52E+00	GT33-B	8.93E+00
GT53-u	GT-C	GT-C	GT-C	Yes	GT83-C	3.33E-03	GT87-C	4.88E-03	GT87-C	5.30E-02
GT69-u	GT-A	-	GT-A	Yes	GT62-A	3.06E-05	GT15-A	8.09E-01	GT60-A	1.02E+00
GT71-u	GT-A	-	GT-A	Yes	GT8-A	1.71E-03	GT24-A	1.10E-01	GT32-A	8.28E-01

GT73-u	variant	-	none	Yes	GT57-C	1.54E+00	GT33-B	6.92E+00	GT16-A	7.37E+00
GT74-u	variant	-	GT-A	No	GT2-A	7.00E-21	GT45-A	1.25E-17	GT27-A	9.35E-17
GT76-u	GT-C	-	GT-C	Yes	GT39-C	2.55E-05	GT50-C	6.59E-04	GT83-C	1.77E-03
GT89-u	GT-C	-	GT-C	Yes	GT83-C	1.86E-03	GT66-C	1.96E-02	GT39-C	3.02E-01
GT91-u	novel	-	none	Yes	GT75-A	3.37E+00	GT16-A	5.89E+00	GT20-B	1.00E+01
GT92-u	GT-A	-	none	No	GT6-A	1.60E+00	GT17-A	2.01E+00	GT55-A	2.51E+00
GT95-u	GT-A	-	none	No	GT6-A	2.23E+00	GT16-A	2.81E+00	GT65-B	3.33E+00
GT96-u	novel	-	none	Yes	GT6-A	5.24E-02	GT34-A	1.68E-01	GT8-A	3.72E-01
GT98-u	GT-C	-	GT-C	Yes	GT66-C	1.22E-07	GT6-A	3.03E+00	GT32-A	5.63E+00
GT99-u	variant	variant	GT-B	No	GT19-B	3.00E-02	GT25-A	1.01E-01	GT30-B	2.58E-01

Supplementary Table 3: A comparison of CNN-attention model with other state of the art models. The comparison is performed on same training dataset as CNN-attention model. Three compared methods are Transformer embeddings with GBDT classifier, single layer LSTM model, ProtCNN model. The results are analysis based on four aspects, fold level accuracy, family level accuracy, interpretability and the ability to classify GT-u families. The interpretability and ability to handle unknown fold sequences were also compared across models. The CNN-attention model has interpretable outputs for every step compared to the Transformer+GBDT model which provides some clustering results based on the projections but does not provide any information about core conserved features, while the LSTM and ProtCNN methods do not provide any such interpretable outputs. Because we incorporate autoencoder models, GT CNN-attention is able to handle unknown fold sequences, whereas other methods cannot.

Model	Fold Accuracy	Family Accuracy	Interpretability	Classify GT-u
**CNN-attention(ours)**	**0.96**	**0.77**	**Yes**	**Yes**
Transformer+GBDT	0.96	0.67	Limited	No
LSTM	0.47	0.34	No	No
ProtCNN	0.84	0.76	No	No

Supplementary Table 4: List of GT families and their corresponding fold and cluster. A Gaussian Mixture Model (GMM) was used to cluster families based on their 2D UMAP projections generated separately for each fold type. Families with a GMM score above -7.5 for GT-A, -7 for GT-B and -6.5 for GT-C were placed in a cluster.

Fold	Cluster	Families	GMM-Score
GT-A	GT-A0	GT16	-5.4641
		GT2	-5.524542
		GT60	-5.642016
		GT14	-5.688812
		GT45	-5.694006
		GT25	-5.785815
		GT78	-5.786725
		GT49	-5.945889
		GT21	-6.060102
		GT27	-6.150723
		GT84	-6.30725
		GT13	-6.346881
		GT24	-6.38189
		GT81	-6.477958
		GT8	-6.500215
		GT32	-6.599175
		GT12	-7.134441
	GT-A1	GT31	-5.309125
		GT15	-5.494145
		GT17	-5.566161
		GT7	-5.657536
		GT77	-5.716002
		GT43	-5.777749
		GT34	-5.940145
		GT67	-5.945407
		GT62	-6.155893
		GT6	-6.906416
	GT-A-Ungrouped	GT88	-7.635158
		GT64	-8.277095
		GT54	-8.51051
		GT82	-9.230959
		GT55	-9.308303
		GT40	-10.256429
		GT75	-11.761891
GT-B	GT-B0	GT9	-4.539272
		GT90	-4.614016
		GT72	-4.620983
		GT93	-4.639714
		GT1	-4.824178
		GT4	-4.83733
		GT63	-4.837775
		GT79	-4.847818

		GT38	-4.904147
		GT10	-5.109297
		GT20	-5.238405
		GT37	-5.267101
		GT28	-5.505519
		GT47	-5.912927
		GT5	-5.96545
		GT61	-6.830745
	**GT-B1**	GT41	-5.310586
		GT3	-5.656573
		GT35	-5.760154
		GT23	-6.101421
		GT19	-6.834921
	**GT-B2**	GT65	-6.139725
		GT104	-6.484054
		GT30	-6.673493
		GT56	-6.829993
		GT80	-6.840005
		GT70	-6.889586
	**GT-B-Ungrouped**	GT33	-7.144054
		GT18	-7.231676
		GT94	-7.454821
		GT52	-8.463694
		GT68	-8.781483
**GT-C**	**GT-C0**	GT39	-5.025598
		GT66	-5.590182
		GT57	-5.851775
	**GT-C1**	GT87	-4.413031
		GT58	-5.296014
		GT50	-6.05887
	**GT-C2**	GT22	-5.371296
		GT83	-5.807555
	**GT-C-Ungrouped**	GT85	-6.935044
		GT59	-8.834369
**GT-lyso**	**GT-lyso**	GT51	-

Supplementary Table 5: Fold prediction results for the GT-u families. RE against all known GTs are shown. RE < 0.107 (Upper 95% CI) suggesting families most likely to adopt a known GT fold are highlighted in yellow and RE > 0.127 (Closer to or more than upper 99% CI) for families most likely to adopt a novel fold are highlighted in red. GT-u families are most likely to adopt the GT fold with the highest positive FAS. Families predicted to adopt a variant or a novel fold have negative FAS scores for all the clusters. The highest FAS scores for families predicted to adopt known folds are colored in green. The predicted fold and confidence are indicated in the last two columns. Confidence is evaluated based on the RE and FAS scores.

Family	Reconstruction Error RE (against All known GTs)	Fold Assignment Scores (FAS)										Prediction Confidence	Predicted fold
		GT-A0	GT-A1	GT-B0	GT-B1	GT-B2	GT-C0	GT-C1	GT-C2	GT-lyso	Max FAS score		
GT53-u	0.0848	-7.7062	-17.1023	-20.4061	-3.4586	-11.7955	1.6505	7.7203	2.6355	-29.5924	7.7203	High	GT-C
GT105-u	0.0850	-17.5652	-24.5877	-27.2568	-6.0943	-17.2715	-3.7203	2.2301	-10.6613	-43.8454	2.2301	High	GT-C
GT110-u	0.0874	0.5617	2.1336	1.6885	-2.1761	0.8386	-28.8364	-41.8105	-26.1506	-10.5905	2.1336	High	GT-A
GT89-u	0.0913	-1.6840	-4.6917	-5.6962	0.8086	-3.2460	1.7864	2.8052	6.1896	-13.9826	6.1896	High	GT-C
GT76-u	0.0914	-19.0593	-26.4973	-29.2158	-5.7049	-20.1115	-4.4119	2.8857	-10.5468	-36.8289	2.8857	High	GT-C
GT106-u	0.0930	-0.8764	0.2566	1.0686	-0.3207	-0.8118	-19.1842	-27.1105	-15.6718	-10.9195	1.0686	High	GT-B
GT109-u	0.0960	0.1085	0.5548	0.2431	0.2316	-1.3323	-20.6968	-30.3417	-19.6562	-14.0250	0.5548	Medium	GT-A
GT29-u	0.0981	-1.7044	0.4057	-0.0964	-4.1254	-3.9635	-34.3220	-44.1672	-32.0038	-15.6071	0.4057	Medium	GT-A
GT103-u	0.1023	-0.9478	-0.3071	-0.1846	0.2727	0.5684	-17.7835	-25.6814	-10.9217	-11.8482	0.5684	Medium	GT-B
GT107-u	0.1026	-1.7008	-0.3479	1.0348	0.1481	1.5433	-20.0356	-24.7787	-12.7447	-9.4429	1.5433	Medium	GT-B
GT69-u	0.1028	-0.2249	1.3810	-1.5294	-3.4850	-3.3949	-30.2525	-42.1769	-27.9236	-11.6703	1.3810	Medium	GT-A
GT102-u	0.1041	-2.8304	-2.1481	0.8966	-1.3502	-0.2873	-32.6477	-42.2431	-28.8707	-23.9149	0.8966	Medium	GT-B
GT71-u	0.1042	-0.6198	0.5227	-1.1886	-0.4666	-1.2484	-16.2671	-24.2920	-11.2713	-7.6293	0.5227	Medium	GT-A
GT98-u	0.1077	-3.5928	-7.0913	-9.1323	-0.5953	-4.5628	1.3219	2.3093	6.5837	-21.9022	6.5837	Low	GT-C
GT92-u	0.1164	-1.0359	0.6344	-4.7917	-7.1016	-5.0746	-37.4749	-49.0855	-32.7090	-13.6905	0.6344	Low	GT-A
GT95-u	0.1138	-1.6167	0.2050	-2.2480	-4.9121	-6.2884	-35.9600	-49.1779	-31.2102	-15.7293	0.2050	Low	GT-A
GT42-u	0.1073	-2.3182	-1.9045	-0.9173	-2.9590	-5.5915	-34.2709	-47.3138	-33.2359	-21.2766	-0.9173	Low	Variant
GT101-u	0.1117	-3.4019	-2.5153	-0.7002	-2.5644	-4.1142	-35.4129	-47.4813	-30.8002	-26.3136	-0.7002	Low	Variant
GT11-u	0.1143	-3.9747	-3.8743	-1.6070	-5.9780	-7.9233	-49.7788	-64.5324	-46.9072	-35.2588	-1.6070	Medium	Variant
GT44-u	0.1148	-4.4052	-7.1966	-3.8032	-1.8888	-9.5213	-27.6446	-36.4394	-27.4815	-31.9549	-1.8888	Medium	Variant
GT100-u	0.1152	-4.2930	-3.2511	-1.1603	-5.2216	-5.5395	-46.6134	-60.9087	-41.1213	-31.2184	-1.1603	Medium	Variant
GT48-u	0.1164	-2.1581	-4.3775	-4.5470	-0.0394	-4.3777	-8.9187	-14.8223	-4.6367	-16.7056	-0.0394	Medium	Variant
GT99-u	0.1201	-4.0847	-3.5176	-0.4866	-2.8529	-4.2945	-37.0718	-47.9242	-36.3760	-28.2846	-0.4866	High	Variant
GT74-u	0.1206	-1.4762	-0.6952	-3.8812	-5.7720	-6.6236	-39.2722	-58.2878	-35.5920	-21.4848	-0.6952	High	Variant
GT73-u	0.1254	-3.3259	-1.7022	-2.3812	-6.0193	-8.2526	-46.8867	-61.8775	-42.3077	-27.5473	-1.7022	High	Variant
GT96-u	0.1302	-2.0410	-0.3352	-3.1068	-2.1614	-2.6451	-22.3939	-29.5370	-17.4108	-11.4877	-0.3352	Low	Novel
GT26-u	0.1323	-4.7941	-7.0916	-0.9723	-5.9388	-8.4612	-43.8379	-61.4936	-45.9031	-40.3355	-0.9723	Low	Novel
GT91-u	0.1360	-2.2260	-0.9271	-7.7585	-6.2706	-5.0966	-31.1590	-36.7761	-23.2723	-7.6507	-0.9271	Medium	Novel
GT97-u	0.1464	-7.6683	-6.8926	-1.3011	-7.7251	-8.8101	-62.2334	-77.3602	-63.1995	-44.5282	-1.3011	Medium	Novel
GT108-u	0.2819	-12.5744	-11.9952	-27.6863	-34.7669	-32.2872	-94.3019	-131.0731	-93.7474	-66.5097	-11.9952	High	Novel
