# Supplementary material for: Mapping the glycosyltransferase fold landscape using interpretable deep learning
Source: Nat Commun. 2021 Sep 27;12:5656. doi: 10.1038/s41467-021-25975-9 (PMC8476585; doi:10.1038/s41467-021-25975-9)
Supplement: Supplementary file 3 — Description of Additional Supplementary Files [file 41467_2021_25975_MOESM3_ESM.pdf]

**Description of Additional Supplementary Files**

File Name: Supplementary  Data 1
Description: List of GenBank IDs for all the GT sequences used in this study.
