# Supplementary material for: Mapping the glycosyltransferase fold landscape using interpretable deep learning
Source: Nat Commun. 2021 Sep 27;12:5656. doi: 10.1038/s41467-021-25975-9 (PMC8476585; doi:10.1038/s41467-021-25975-9)
Supplement: Supplementary file 5 — Reporting Summary [file 41467_2021_25975_MOESM5_ESM.pdf]

Corresponding author(s): Natarajan Kannan

Last updated by author(s): Aug 11, 2021

Reporting Summary

Nature Portfolio wishes to improve the reproducibility of the work that we publish. This form provides structure for consistency and transparency in reporting. For further information on Nature Portfolio policies, see our Editorial Policies and the Editorial Policy Checklist.

Statistics

For all statistical analyses, confirm that the following items are present in the figure legend, table legend, main text, or Methods section.

n/a	Confirmed	
☐	☒	The exact sample size (n) for each experimental group/condition, given as a discrete number and unit of measurement
☐	☒	A statement on whether measurements were taken from distinct samples or whether the same sample was measured repeatedly
☐	☒	The statistical test(s) used AND whether they are one- or two-sided<br>*Only common tests should be described solely by name; describe more complex techniques in the Methods section.*
☐	☒	A description of all covariates tested
☐	☒	A description of any assumptions or corrections, such as tests of normality and adjustment for multiple comparisons
☐	☒	A full description of the statistical parameters including central tendency (e.g. means) or other basic estimates (e.g. regression coefficient) AND variation (e.g. standard deviation) or associated estimates of uncertainty (e.g. confidence intervals)
☐	☒	For null hypothesis testing, the test statistic (e.g. F, t, r) with confidence intervals, effect sizes, degrees of freedom and P value noted<br>*Give P values as exact values whenever suitable.*
☐	☒	For Bayesian analysis, information on the choice of priors and Markov chain Monte Carlo settings
☐	☒	For hierarchical and complex designs, identification of the appropriate level for tests and full reporting of outcomes
☐	☒	Estimates of effect sizes (e.g. Cohen's d, Pearson's r), indicating how they were calculated

Our web collection on statistics for biologists contains articles on many of the points above.

Software and code

Policy information about availability of computer code

Data collection	The IDs for the protein sequences used in this study were directly collected from the CAZy database and these IDs were used to collect the sequences from the NCBI database using the Batch Entrez portal. No additional software was used for the collection of these sequences.
Data analysis	Secondary structure prediction was conducted using NetSurfP2.0. NCBI Batch CD-search tool was used to determine the domain bounds. Processing of sequences, training and use of deep learning model, calculation of scores and the generation of plots and images was performed using custom code written in python3.7 with the sklearn 0.24.1, pytorch v1.8+ and cuda v11.1 packages. The code, along with all related datasets are available at https://doi.org/10.5281/zenodo.5173136.

For manuscripts utilizing custom algorithms or software that are central to the research but not yet described in published literature, software must be made available to editors and reviewers. We strongly encourage code deposition in a community repository (e.g. GitHub). See the Nature Portfolio guidelines for submitting code & software for further information.

Data

Policy information about availability of data

All manuscripts must include a data availability statement. This statement should provide the following information, where applicable:

- Accession codes, unique identifiers, or web links for publicly available datasets
- A description of any restrictions on data availability
- For clinical datasets or third party data, please ensure that the statement adheres to our policy

For the sequences used in this study, the IDs were collected from the CAZy database (http://www.cazy.org/GlycosylTransferases.html) and using those IDs, the sequences were obtained from the NCBI database (https://www.ncbi.nlm.nih.gov/protein/) using batch Entrez (https://www.ncbi.nlm.nih.gov/sites/batchentrez). All the sequences and their secondary structure predictions that were used for training and testing both the CNN-attention and the autoencoder models have been

made available through https://github.com/esbgkannan/GT-CNN. The sequence and secondary structure prediction data used in this study are available in the Zenodo database under the following DOI: https://doi.org/10.5281/zenodo.5173136. Source data are provided with this paper.

Field-specific reporting

Please select the one below that is the best fit for your research. If you are not sure, read the appropriate sections before making your selection.

☒ Life sciences ☐ Behavioural & social sciences ☐ Ecological, evolutionary & environmental sciences

For a reference copy of the document with all sections, see nature.com/documents/nr-reporting-summary-flat.pdf

Life sciences study design

All studies must disclose on these points even when the disclosure is negative.

Sample size	All available GT sequences listed in the CAZy datanase were downloaded and filtered for sequence similarity and used for training and testing the model.
Data exclusions	No data were excluded from the analyses.
Replication	The U-MAP projection was generated across a combination of parameters with 3 replicates for each combination. Replicates generated similar projections.
Randomization	For training the CNN-attention model, the randomized dataset was generated using stratified sampling (https://scikit-learn.org/stable/modules/generated/sklearn.model_selection.train_test_split.html) based on the family level of GTs with a split of train (141984):test (1643): val(1643). For the autoencoder model, the split was train (129498):test (948): val(948).
Blinding	Blinding is not relevant to the study since the researchers provide all the sequences with their labels for a supervised training of the deep learning model.

Reporting for specific materials, systems and methods

We require information from authors about some types of materials, experimental systems and methods used in many studies. Here, indicate whether each material, system or method listed is relevant to your study. If you are not sure if a list item applies to your research, read the appropriate section before selecting a response.

Materials & experimental systems

n/a	Involved in the study	
☒	☐	Antibodies
☒	☐	Eukaryotic cell lines
☒	☐	Palaeontology and archaeology
☒	☐	Animals and other organisms
☒	☐	Human research participants
☒	☐	Clinical data
☒	☐	Dual use research of concern

Methods

n/a	Involved in the study	
☒	☐	ChIP-seq
☒	☐	Flow cytometry
☒	☐	MRI-based neuroimaging
